# Role of Lung Ultrasonography (LUS) as a Tool for Evaluating Children with Pediatric Inflammatory Multisystem Syndrome Temporally Associated with SARS-CoV-2 (PIMS-TS)

**DOI:** 10.3390/jcm12082850

**Published:** 2023-04-13

**Authors:** Jolanta Tomczonek-Moruś, Natalia Krysiak, Agnieszka Blomberg, Marta Depczyk-Bukała, Marcin Tkaczyk, Krzysztof Zeman

**Affiliations:** 1 Department of Pediatrics, Immunology and Nephrology, Institute of the Polish Mother’s Memorial Hospital in Lodz Poland, 93-338 Lodz, Poland; 2 Department of Pediatrics Nephrology and Immunology, Medical University of Lodz, 90-151 Lodz, Poland

**Keywords:** lung ultrasound, PIMS-TS, MIS-C, pneumonia, intensive care, children, adolescents

## Abstract

Background: Pediatric inflammatory multisystem syndrome temporally associated with SARS-CoV-2 (PIMS-TS) is a novel entity. The inflammatory process involves the circulatory, digestive, respiratory, and central nervous systems, as well as the skin. Making a diagnosis requires extensive differential diagnoses, including lung imaging. The aim of our study was to retrospectively assess the pathologies found in lung ultrasound (LUS) in children diagnosed with PIMS-TS and to evaluate the usefulness of the examination in diagnostics and monitoring. Methods: The study group consisted of 43 children diagnosed with PIMS-TS, in whom LUS was performed at least three times, including on admission to hospital, on discharge, and 3 months after disease onset. Results: Pneumonia (mild to severe) was diagnosed in 91% of the patients based on the ultrasound image; the same number had at least one pathology, including consolidations, atelectasis, pleural effusion, and interstitial or interstitial-alveolar syndrome. By the time of discharge, the inflammatory changes had completely regressed in 19% of the children and partially in 81%. After 3 months, no pathologies were detected in the entire study group. Conclusion: LUS is a useful tool for diagnosing and monitoring children with PIMS-TS. Inflammatory lesions of the lungs resolve completely when the generalized inflammatory process subsides.

## 1. Introduction

In 2019 reports of a previously unknown set of clinical symptoms appeared in the pediatric population. An association with SARS-CoV-2 was observed. On 7 April 2020, the first case of a novel entity was registered, called multisystem inflammatory syndrome in children (MIS-C) or adolescents (MIS-A) by the United States Centers for Disease Control (CDC) and the World Health Organization (WHO). At the same time, the Royal College of Pediatrics and Child Health (RCPCH) used the term pediatric inflammatory multisystem syndrome temporally associated with SARS-CoV-2 (PIMS-TS), with slightly different criteria [1,2,3]. We will use this name in our article, but both terms function in parallel in the literature. It is coded with ‘U10’ in the International Classification of Diseases [4].

Symptoms of PIMS-TS appear 4–8 weeks after SARS-CoV-2 infection and are the result of the immune system’s reaction to the infectious agent. Cytokine-dependent inflammation of small vessels occurs. The leading symptom of PIMS-TS is fever. Children also present manifestations from the digestive system (abdominal pain, vomiting, or diarrhea), the cardiovascular system (tachycardia, low-output syndrome, or coronary artery aneurysm), the central nervous system (headache, photophobia, meningeal syndrome, or hyperaesthesia), the respiratory system (cough, shortness of breath, or respiratory failure) or the skin and mucous membranes (erythema, exanthema, or aphthae). The general condition of patients is moderate or severe, often requiring treatment in the pediatric intensive care unit due to circulatory and/or respiratory failure. The variety of clinical symptoms and their concurrence, as well as the lack of specific laboratory markers, necessitates a thorough and extensive differential diagnosis. According to the current criteria, making a diagnosis of PIMS-TS requires that other infectious and non-infectious causes be excluded, e.g., sepsis, toxic shock syndrome (TSS), meningitis, acute abdominal diseases, pneumonia, myocarditis, or other multi-organ inflammatory diseases, such as hemophagocytic syndrome or Kawasaki disease [5,6,7,8,9,10,11,12,13]. It requires many laboratory and imaging tests confirming the involvement of at least two systems. There are still few articles focussing on the analysis of lung imaging findings and their evolution in the acute state, and even fewer on lung residues after PIMS-TS.

In the last decade, the role of ultrasound in assessing the lungs and pleura in children has increased [14]. This tool is easily accessible, repeatable, and—compared to X-ray—safer, more sensitive, and specific in the imaging of inflammatory lung changes and pleural effusion [15]. Incomplete skeletal ossification in children and the typically sparse adipose tissue make it possible to use additional acoustic windows [14].

The aim of our study was to evaluate pathologies on LUS in children with PIMS-TS and to assess the usefulness of this examination in the diagnosis pathway and monitoring.

## 2. Materials and Methods

The study was conducted from November 2019 to March 2022 among patients hospitalized at the Department of Pediatrics, Immunology, and Nephrology of the Polish Mother’s Memorial Hospital Research Institute in Łódź, Poland. We retrospectively evaluated the lung ultrasound (LUS) images of all patients hospitalized with fever for more than 3 days and symptoms associated with PIMS-TS from at least two systems. The examinations were performed with a linear or curvilinear probe (Philips Affiniti 70G eL18-4 and C8-2, respectively) by only four pediatric specialists with certified LUS training and extensive experience in ultrasound imaging. After use, all materials were disinfected correctly.

The examination included the assessment of the entire lung fields on the anterior, lateral, and posterior surfaces of the chest and in the sagittal and frontal planes. LUS was carried out at least twice—in all patients on the first day after admission and before discharge—but most had it performed 3–4 times, depending on their clinical condition.

Inflammatory lesions of the lungs were diagnosed on the basis of applicable parenchymal, pleural, and vascular criteria [15,16,17,18]. They were classified as B-line artifacts, interstitial syndrome, interstitial-alveolar syndrome, consolidations, and atelectatic lesions. The presence of fluid in the pleural cavity was also assessed, typically in the sitting position, unless the patient’s clinical condition did not allow it, in which case it was done in the lateral or supine position (e.g., in ICU patients). In our study, we estimated the pleural effusion layer in millimeters but not in total volume. A normal ultrasound image of the lungs was classified as the presence of all the following criteria: sliding sign visible over the entire surface of the lungs, A-line artifacts visible over the entire surface of the lungs, a continuous, smooth pleural line over the entire surface of the lungs, no consolidation, no pleural effusion, a visible layer of fluid <2 mm, and single (1–2) B-line artifacts at the base of the lungs. No LUS score was used to assess the severity of lung involvement.

LUS was repeated in all patients before hospital discharge and 3 months after discharge. The assessment was made according to the same methodology as given above. All study participants underwent a typical physical examination of the respiratory system on each day of hospitalization. Routine chest radiographs were not performed, as this was not the subject of our analysis.

The study was approved by our institute’s ethics committee (No. 45/2021). Parental consent for the study was obtained.

## 3. Results

**Figure 1:**
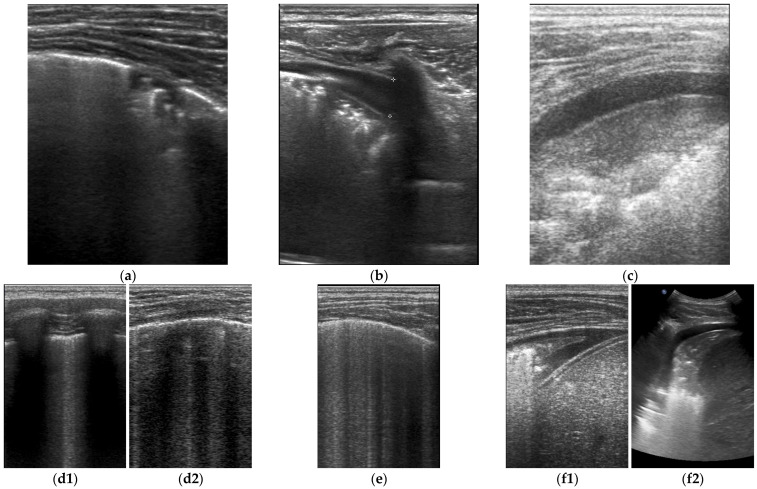
LUS images (**a**)—consolidation; (**b**,**c**)—consolidation and fluid in pleural cavity; (**d1**)—interstitial syndrome—a longitudinal projection; (**d2**)—interstitial syndrome—a transversal projection; (**e**)—interstitial-alveolar syndromes; (**f1**)—atelectasis with consolidation and pleural effusion/linear probe/; (**f2**)—atelectasis with consolidation and pleural effusion/curvilinear probe/.

In the given period, 47 children met the criteria for a PIMS-TS diagnosis, and 43 met all evaluation criteria (30 boys and 13 girls, aged from 11 months to 17 years 4 months; average: 6 years 7 months; median: 6 years). Abnormalities on physical examination were found in 21 subjects (49%). These included tachypnoea and asymmetry of respiratory murmurs or crackles. 39 of the 43 patients (91%) were diagnosed with pneumonia based on ultrasound criteria. The same number of children (39/43; 91%) had more than one pathology, and 28 of them (65%) had bilateral lesions. The most common pathologies were consolidations with air bronchogram or mixed bronchogram, which were observed in 37 respondents (86%) [Figure 1a]. These were followed, in descending order, by fluid in the pleura (36/43 [84%]) in the amount of 2 to 55 mm (average 11.5 mm: median 24.3 mm) [Figure 1b,c], interstitial syndrome (30/43 [70%]) [Figure 1(d1,d2), atelectasis (28/43 [65%]) [Figure 1(f1,f2)], and interstitial-alveolar syndrome (19/43 [44%]) [Figure 1e]. For other abnormalities, an image of so-called ‘white lungs’ was observed in five patients (12%). A normal LUS image was found in only three of the children (7%). By the day of discharge, total regression of inflammatory changes was observed in eight (19%) and partial regression in 35 (81%) patients. Three months after discharge from the hospital, a normal ultrasound image of the lungs was found in all respondents (43/43 [100%]) (Table 1 and Table 2).

## 4. Discussion

Inflammatory lung changes are one of the diagnostic criteria for PIMS-TS. In our study, on the basis of USG criteria, inflammatory changes in the lungs were diagnosed in the majority of patients (91%), while respiratory symptoms were present in less than half of them (49%). Daniel A. Lichtenstein promoted LUS and proved its superiority over lung auscultation and classical X-ray diagnostics in acute respiratory failure (ARDS) in adults almost 20 years ago [19]. In recent years, LUS has also been used in the pediatric population [14]. A meta-analysis of studies involving a group of 1510 children showed that LUS has similar sensitivity and specificity to chest radiographs in the diagnosis of inflammatory changes in the lungs of children [20].

Part of the chest ultrasound examination is also pleural imaging and evaluation of the presence of fluid in the pleural cavities. LUS is characterized by 100% sensitivity and 97.7% specificity, while chest radiography demonstrates 71% and 88%, respectively [15]. So far, CT remains the gold standard in lung imaging. Due to the impact of ionizing radiation, as well as high operating and maintenance costs, it should not be performed routinely. In addition, in younger children, CT usually requires sedation. Moreover, studies comparing LUS and chest CT in the assessment of inflammatory lung lesions in children are not yet known.

As mentioned in the introduction, the clinical picture of MIS-C/PIMS-TS is quite heterogeneous. With the exception of fever, which is the leading symptom, the symptoms occur with different coincidences, and the diagnosis requires a broad differential diagnosis. Due to the heterogeneity of the clinical signs and symptoms, diagnosis of PIMS-TS is difficult. Based on a systematic review from 2021, it was found that in the course of PIMS-TS, cough occurred in almost 24% of patients and dyspnea in 26.7%. Respiratory symptoms were not the leading ones, as cardiac symptoms occurred in almost 80% of patients, and abdominal symptoms presented in more than 70% [21]. In our work, 21 children (49%) had abnormalities on physical examination of the respiratory system (tachypnoea, asymmetry of breath murmur, or crackles). Although almost half of the patients with PIMS-TS did not show any respiratory symptoms, most of them were found to have abnormalities on LUS.

The first reported use of LUS in PIMS-TS patients was an Italian paper published in early 2022. Musolino et al. found pathologies in LUS in 10/10 patients (100%) with PIMS-TS, most often as an abnormal pleural image and disseminated B-line artifacts, in 8/10 (80%) interstitial syndrome was visible, in 7/10 (70%) there were subpulmonary consolidations, and in half of the subjects, interstitial-alveolar changes were found. It is worth mentioning that symptoms such as cough or shortness of breath were found in less than half of the respondents (4/10 [40%]) [22]. The results of this work strongly indicate that even in the absence of clinical signs, the lungs are usually involved in the inflammatory process during PIMS-TS. The limitations of that publication are its small study group and lack of follow-up.

In another publication, only 2/24 children (8%) had a normal ultrasound image of the lungs [23]. Another work using point-of-care lung ultrasound (PoCUS-LUS) found pleural fluid in 6/9 subjects (67%) with PIMS-TS [24]. In our publication, abnormal LUS images were found in 91% of the subjects. Compared to the cited publications, ours included a larger group—43 respondents. These reports show that the majority of children with PIMS-TS had pathologies in LUS, while less than 30% of them presented symptoms from the respiratory system [21].

These data differ from classical radiological examinations. Depending on the methodology and the size of the study group, normal chest X-ray images were found in 16–64% of subjects with PIMS-TS [25,26,27]. The systematic review by Hoste et al. reported that disseminated radiological changes were found in 35% of respondents [21]. Even larger discrepancies were found while analyzing chest CT scans. Abnormalities were found in 39–83% of patients, predominantly (>80%) the appearance of milky glass and the presence of consolidation. Pleural effusion was detected in 30–58%, while atelectasis was in 26% [25,26,28].

In our study group, we did not routinely perform chest radiograms. X-rays or CTs were only used for clinical indications, for example, in patients in serious condition or to assess the position of the vascular catheter. Based on our own work and the publications cited herein, it seems that there are currently no indications for routine X-rays or CTs for respiratory assessment in children with PIMS-TS (too much discrepancy between clinic and imaging). Examination by LUS provides quick, repeatable diagnostic information at the bedside that complements physical examination. However, as with other lung imaging modalities, some knowledge and skill are required to use lung ultrasound. If the team is inexperienced in performing lung ultrasounds, the choice of classic chest X-ray seems obvious. Furthermore, accurate imaging of the mediastinum, especially in older and obese children, is impossible. Our team suggests consideration for chest CTs in patients with respiratory failure requiring intubation, with extensive inflammatory changes in the lungs, or in patients with an unclear clinical picture.

An advantage of our study is the fairly large, age-diverse, and clinically heterogeneous study group. We found that the inflammatory lesions of the lungs regressed totally or significantly in all children after effective immunosuppressive treatment. Often, an abnormal LUS image—especially involving bilateral inflammatory lesions with pleural effusion in a patient with fever and in moderate or severe overall condition—directed our diagnostics towards PIMS-TS despite the absence of signs and/or symptoms suggesting respiratory involvement. At the same time, with an ambiguous clinical picture, the use of LUS allowed for a faster diagnosis of PIMS-TS. Currently, it is not known whether similar changes in the lungs are observed in other acute multisystem, hyperinflammatory diseases, such as Kawasaki disease or hemophagocytic syndrome [5,6,22]. So far, there are no data on how long LUS changes persist in the course of PIMS-TS. During our observation, 3 months after discharge from the hospital, normal LUS imagery was found in all patients. From these observations, we conclude that inflammatory changes in the lungs in the course of PIMS-TS are acute.

## 5. Conclusions

LUS, despite its limitations, is a useful tool that can be easily used in the diagnosis, differentiation, and monitoring of inflammatory changes in the lungs. Ultrasound pathologies occur in most patients with PIMS-TS in the acute stage and regress completely with the extinction of the generalized inflammatory process. Ultrasound pathologies and inflammatory changes in the lungs are present in most patients with PIMS-TS, even if respiratory symptoms and signs are absent. In our team, we agree on the need to disseminate LUS as a ‘point-of-care’ in children. As PIMS-TS is still a new entity, patients should be carefully followed up.

## Figures and Tables

**Table 1:** Study group.

**age (years), mean (median)**	**6 7/12 (6)**
**sex, *n* (%)**	
FEMALE	13 (30)
MALE	30 (70)
**respiratory abnormalities signs *n* (%)**	21 (49)
**pneumonia criteria *n* (%)** **on admission**	39 (91)
**>1 pathology *n* (%)**	39 (91)
bilateral lesions, *n* (%)	28 (65)
Normal LUS image, *n* (%)	3 (7)
**by the day of discharge**	
Total regression, *n* (%)	8 (19)
Partial regression, *n* (%)	35 (81)

**Table 2:** LUS findings.

	1-SIDED (TOT 43; %)	2-SIDED (TOT 43; %)	TOTAL (TOT 43; %)	AFTER 3 MONTHS
**B-Lines Artifacts**	0 (0)	40 (93)	40 (93)	0 (0)
**Interstitial Syndrome**	0 (0)	30 (70)	30 (70)	0 (0)
**Interstitial-alveolar syndrome**	0 (0)	19 (44)	19 (44)	0 (0)
**Consolidations**	7 (16)	30 (70)	37 (86)	0 (0)
**Atelectasis**	9 (21)	19 (44)	28 (65)	0 (0)
**Pleural effusion**	0 (0)	36 (84)	36 (84)	0 (0)

## Data Availability

The data that support the findings of this study are available from the corresponding author upon reasonable request.
